# Different Risks of Mortality and Longitudinal Transition Trajectories in New Potential Subtypes of the Preserved Ratio Impaired Spirometry: Evidence From the English Longitudinal Study of Aging

**DOI:** 10.3389/fmed.2021.755855

**Published:** 2021-11-11

**Authors:** Di He, Yilan Sun, Musong Gao, Qiong Wu, Zongxue Cheng, Jun Li, Yong Zhou, Kejing Ying, Yimin Zhu

**Affiliations:** ^1^Department of Respiratory Diseases, Sir Run Shaw Hospital Affiliated to School of Medicine, Zhejiang University, Hangzhou, China; ^2^Department of Epidemiology and Biostatistics, School of Public Health, Zhejiang University, Hangzhou, China; ^3^Department of Respiratory and Critical Care Medicine, Zhejiang Provincial People's Hospital, People's Hospital of Hangzhou Medical College, Hangzhou, China

**Keywords:** preserved ratio impaired spirometry, lung function, heterogeneity, subtypes, mortality, longitudinal transition

## Abstract

**Background:** Preserved ratio impaired spirometry (PRISm), characterized by the decreased forced expiratory volume in 1 s (FEV_1_) and forced vital capacity (FVC) with a preserved FEV_1_/FVC ratio, is highly prevalent and heterogeneous. We aimed to identify the subtypes of PRISm and examine their differences in clinical characteristics, long-term mortality risks, and longitudinal transition trajectories.

**Methods:** A total of 6,616 eligible subjects were included from the English longitudinal study of aging. Two subtypes of the PRISm were identified as mild PRISm (either of FEV_1_ and FVC <80% predicted value, FEV_1_/FVC ≥0.7) and severe PRISm (both FEV_1_ and FVC <80% predicted values, FEV_1_/FVC ≥0.7). Normal spirometry was defined as both FEV_1_ and FVC ≥80% predicted values and FEV_1_/FVC ≥0.7. Hazard ratios (HRs) and 95% CIs were calculated by the multiple Cox regression models. Longitudinal transition trajectories were described with repeated spirometry data.

**Results:** At baseline, severe PRISm had increased respiratory symptoms, including higher percentages of phlegm, wheezing, dyspnea, chronic bronchitis, and emphysema than mild PRISm. After an average of 7.7 years of follow-up, severe PRISm significantly increased the risks of all-cause mortality (HR=1.91, 95%CI = 1.58–2.31), respiratory mortality (HR = 6.02, 95%CI = 2.83–12.84), and CVD mortality (HR = 2.11, 95%CI = 1.42–3.13) compared with the normal spirometry, but no significantly increased risks were found for mild PRISm. In the two longitudinal transitions, mild PRISm tended to transition toward normal spirometry (40.2 and 54.7%), but severe PRISm tended to maintain the status (42.4 and 30.4%) or transition toward Global Initiative for Chronic Obstructive Lung Disease (GOLD)2–4 (28.3 and 33.9%).

**Conclusion:** Two subtypes of PRISm were identified. Severe PRISm had increased respiratory symptoms, higher mortality risks, and a higher probability of progressing to GOLD2–4 than mild PRISm. These findings provided new evidence for the stratified management of PRISm.

## Introduction

Preserved ratio impaired spirometry (PRISm) is characterized by the proportional reductions in the forced expiratory volume in 1 s (FEV_1_) and forced vital capacity (FVC) resulting in a normal FEV_1_/FVC ratio, alternately referred to as the restrictive spirometry pattern (1, 2), Global Initiative for Chronic Obstructive Lung Disease (GOLD)-unclassified (3), or nonspecific pattern (4). It is estimated that the prevalence rates of PRISm in adults range from 3.0 to 20.0% worldwide (1–10). Accumulating evidence had shown that PRISm was associated with increased respiratory symptoms (11, 12), functional limitations (13), cardiovascular comorbidity (14, 15), and increased risks of mortality (1, 2, 5). Furthermore, longitudinal transition studies suggested that PRISm was a fluctuating state between normal spirometry and chronic obstructive pulmonary disease (COPD) (5, 16). Therefore, PRISm is becoming an important lung function phenotype in clinical and public health practices.

Despite the increasing concern about PRISm, the disease courses and pathological mechanisms of PRISm are not well understood. Previous studies indicated that PRISm was a heterogeneous phenotype and had no uniform definition (5, 6, 16–18). PRISm was defined as FEV_1_ <80% predicted value with FEV_1_/FVC ≥0.7 (5, 6) or FVC <80% predicted value with FEV_1_/FVC ≥0.7 (1, 15). These two definitions only consider the reduction in single lung function indicator (FEV_1_ or FVC). However, both FEV_1_ and FVC are associated with the risks of adverse health outcomes (19, 20). Therefore, it is reasonable to use both FEV_1_ and FVC to define the lung function reduction in PRISm. Furthermore, there are different degrees of reductions in FEV_1_ and FVC among the PRISm subjects. Some PRISm subjects have a single reduction in FEV_1_ or FVC, whereas others have both reductions in FEV_1_ and FVC. The single reduction and both reductions might have different biological effects, and represent different subtypes of PRISm. Based on the above hypothesis, we, therefore, defined two subtypes of PRISm as mild PRISm (single reduction in FEV_1_ or FVC) and severe PRISm (both reductions in FEV_1_ and FVC).

Using the data of the English Longitudinal Study of Aging (ELSA) cohort, we aimed to identify the two subtypes of PRISm, and examine their differences in clinical characteristics, long-term mortality risks, and longitudinal transition trajectories.

## Materials and Methods

### Study Design and Population

The English Longitudinal Study of Aging is an ongoing and prospective cohort study conducted in the UK. Detailed study designs, methods, and procedures have been described previously (21, 22). Briefly, a representative sample of the community-dwellers aged ≥50 years was recruited in 2002–2003 (Wave 1). Participants were investigated biennially using the self-completion questionnaires and computer-assisted personal interviews. Additional nurse visits were conducted every 4 years for the assessments of anthropometric and biochemical indicators. The baseline of this study was Wave 2 (2004–2005), which included the first nurse visit data combined with the questionnaire data. The ELSA study was approved by the London Multi-Center Research Ethics Committee and informed consent was obtained from each participant.

**Figure 1:**
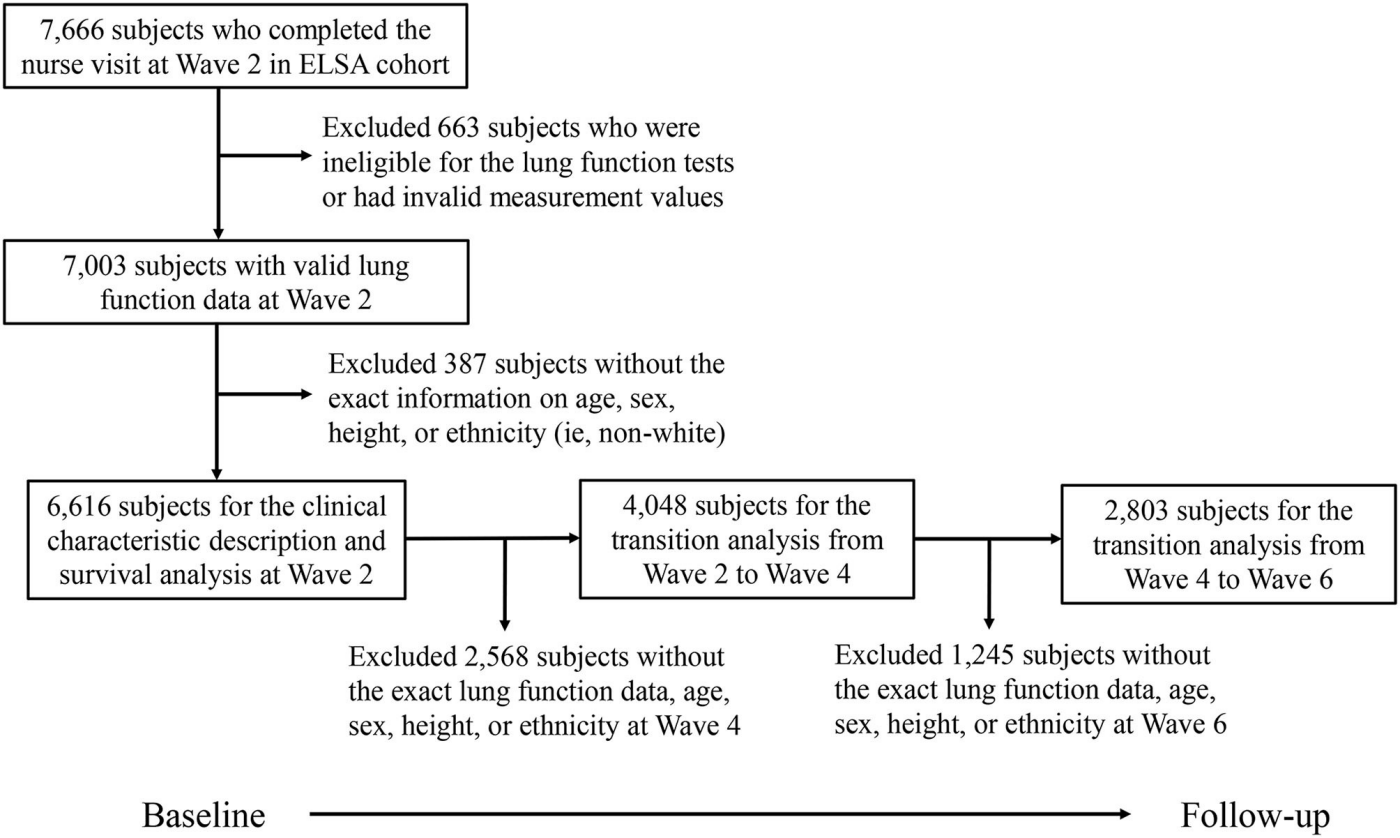
Flowchart of the study population selection procedure. ELSA, English longitudinal study of aging.

Figure 1 describes the selection procedure of the subjects for the present study. Among the 7,666 subjects who completed the first nurse visit, we first excluded 663 subjects who were ineligible for the lung function tests or had invalid measurement values. Then, 387 subjects were further excluded without the exact information on age, sex, height, or ethnicity. Finally, 6,616 subjects were included at baseline for the description of clinical characteristics and survival analysis. In addition, for the analysis of the longitudinal transition from Wave 2 to Wave 4, 2,568 ineligible subjects were excluded, the remaining 4,048 subjects were included in the analysis. Similarly, we excluded 1,245 ineligible subjects, the remaining 2,803 subjects were included for the analysis of the longitudinal transition from Wave 4 to Wave 6.

### Definitions of Lung Function Categories

Prebronchodilator spirometry was carried out using a spirometer following standard procedures based on the American Thoracic Society guideline (23). The values of FEV_1_, FVC, and peak flow (PF) were measured. Before the formal test, each subject was asked to take off any tight clothing and was told that they must try to blow the air as hard as possible. Then, the trained nurse demonstrated the correct operation of spirometry, and each subject was instructed to make some practices. In the formal test, three satisfactory measurements were recorded for each subject. The unsatisfactory measurement was defined as follows: an unsatisfactory start with excessive hesitation; coughing or laughing, especially in the first second; leakage of the air around the mouthpiece; obstruction of the mouthpiece by teeth or tongue; and obstruction of the flow head outlet by hands. The most satisfactory values of FEV_1_, FVC, and PF were used in the final analyses. The most satisfactory values were defined as the maximal FEV_1_, FVC, and PF from the three measurements, no matter which measurement generated the values. Predicted values of FEV_1_, FVC, and the lower limit of normal (LLN) were calculated using the Global Lung Function Initiative 2012 equations according to age, sex, height, and ethnicity (24).

Preserved ratio impaired spirometry was divided into two subtypes as mild PRISm and severe PRISm. The other lung function categories included normal spirometry, GOLD1, and GOLD2–4. Normal spirometry was defined as both FEV_1_ and FVC ≥80% predicted values and FEV_1_/FVC ≥0.7. Mild PRISm was defined as either of FEV_1_ and FVC <80% predicted value and FEV_1_/FVC ≥0.7, while severe PRISm was defined as both FEV_1_ and FVC <80% predicted values and FEV_1_/FVC ≥0.7. GOLD1 and GOLD2–4 were defined based on the modified GOLD criteria: GOLD1 (FEV_1_ ≥80% predicted value and FEV_1_/FVC <0.7); GOLD2–4 (FEV_1_ <80% predicted value and FEV_1_/FVC <0.7) (25). In addition, the five-lung function categories were also defined based on the LLN criteria. In the LLN criteria, normal spirometry was defined as all of FEV_1_, FVC, and FEV_1_/FVC ≥LLN; mild PRISm was defined as either of FEV_1_ and FVC < LLN and FEV_1_/FVC ≥LLN; severe PRISm was defined as both of FEV_1_ and FVC < LLN and FEV_1_/FVC ≥LLN; GOLD1 was defined as FEV_1_ ≥LLN and FEV_1_/FVC < LLN; and GOLD2-4 was defined as FEV_1_ < LLN and FEV_1_/FVC < LLN.

### Assessments of Covariates and Mortality

The covariates in this study included demographic and clinical variables. For demographic variables, education was divided into two levels as less than college and college or above. Marital status was classified as married or partnered and others. Smoking status was divided into three categories, namely, never smokers, former smokers, and current smokers. Former smokers and current smokers were classified as ever smokers. Drinking status was evaluated by the frequency of drinking per week, and was classified as less than one time, one to four times, and greater than or equal to five times. Physical activity level was categorized into three groups, namely, vigorous (vigorous activity greater than one time per week), moderate (moderate activity greater than one time per week), and inactive (the rest). Body mass index (BMI) was defined as the weight (kg) divided by the square of height (m^2^). For clinical variables, the symptoms of wheezing and phlegm were determined by the questions of “whether had attacks of wheezing or whistling in your chest during the last 12 months” and “whether usually brought up phlegm from your chest in the morning, during the day or at night in winter,” respectively. The modified Medical Research Council (mMRC) dyspnea scale was used to assess the severity of breathlessness and dyspnea was defined as the mMRC Grade ≥2 (26, 27). The presences of chronic bronchitis or emphysema, cardio-cerebrovascular disease (CVD), and cancer were based on the self-reported physician diagnosis. Mortality data were obtained from the end of life interviews, which were available up to Wave 6 (2012, 2013). The end of life interviews was applicable to the 6,616 subjects included at baseline. The main cause of death was classified as cancer, respiratory disease, CVD, or other cause.

### Data Imputation and Statistical Analyses

Multiple imputations were used to deal with the missing data of the covariates. The method of the imputation was chained equations according to the original nature of the imputed variable (continuous or categorical). In the present study, we created 10 imputed datasets with 6,616 included subjects. All of the statistical analyses were conducted separately in each of the 10 datasets, and the results were pooled using the R package MICE.

Continuous variables with normal distribution were expressed as mean (SD), while continuous variables with skewed distribution were expressed as median [interquartile range (IQR)]. Categorical variables were expressed as numbers (percentages). Comparisons among the five lung function categories were conducted using the one-way ANOVA or Kruskal–Wallis rank sum tests for continuous variables, and chi-square tests for categorical variables.

To investigate the long-term mortality risks of different lung function categories, multiple Cox regression models were used to calculate the hazard ratios (HRs) and 95% CIs using the normal spirometry or severe PRISm as the reference. The proportional hazard assumption was confirmed using the Schoenfeld residual. The adjusted covariates included age, sex, marital status, education level, BMI, baseline CVD and cancer, smoking status, drinking status, and physical activity level. For the analyses of respiratory mortality and CVD mortality, we additionally excluded 195 subjects due to the missing data on the cause of death. Furthermore, we described the longitudinal transition trajectories of different lung function categories from Wave 2 to Wave 4 and Wave 4 to Wave 6. The number (percentage) of subjects and the median (IQR) of annual changes of FEV_1_ and FVC were calculated for each transition trajectory.

Several sensitivity analyses were performed. We repeated the analyses using the LLN criteria to define the lung function categories. Furthermore, we conducted the survival analyses after excluding subjects with asthma at baseline or stratified by sex and smoking status, respectively.

All the analyses were carried out using R software (Version 4.0.2). All the *P*-values were two-sided. The statistical significance was defined as the *P* <0.05. The borderline significance was defined as the *P* > 0.05, but <0.10 (0.05 ≤ *P* <0.10).

## Results

### Baseline Characteristics of the Subjects

Among the 6,616 eligible subjects at baseline, the mean age was 65.8 years and 3,609 (54.5%) were women. The prevalence rates of mild PRISm and severe PRISm were 7.5 and 12.9%, respectively. Table 1 presents the baseline characteristics of subjects by the different lung function categories. Subjects with GOLD2–4 were the oldest (68.4 ± 9.2 years), had the lowest education level (26.2% of college or above), the lowest percentage of the married or partnered (63.1%), and the highest percentage of current smoking (27.6%), then followed by those with severe PRISm and mild PRISm. Both mild PRISm and severe PRISm groups had higher means of BMI (29.1 ± 5.1 and 28.9 ± 5.4 kg/m^2^) than other lung function categories, and the highest percentage of inactive physical activity (31.8%) was observed in the severe PRISm group. In addition, GOLD2–4 and severe PRISm seemed to have more serious clinical characteristics than normal spirometry, mild PRISm, and GOLD1. Subjects with GOLD2–4 had the lowest means of FEV_1_(1.42 ± 0.61 L), PF (273.4 ± 127.5 L), the highest percentages of phlegm (35.4%), wheezing (33.8%), dyspnea (26.3%), and chronic bronchitis or emphysema (15.0%). Subjects with severe PRISm had the lowest mean of FVC (2.14 ± 0.68 L), the highest median of CRP (3.1, IQR: 1.5–6.1 mg/L), and the highest percentages of CVD (29.2%) and cancer (8.6%).

**Table 1:** Baseline characteristics among subjects by the lung function categories.

	**Normal**	**Mild**	**Severe**	**GOLD1**	**GOLD2–4**	***P* value**
	**spirometry**	**PRISm**	**PRISm**			
Number	3,450	494	852	721	1,099	
Age, year, mean (SD)	64.4 (8.7)	66.3 (9.2)	67.7 (9.3)	66.2 (8.9)	68.4 (9.2)	<0.001
Female, *n* (%)	1,951 (56.6)	268 (54.3)	481 (56.5)	351 (48.7)	558 (50.8)	<0.001
BMI, (kg/m2), mean (SD)	28.0 (4.7)	29.1 (5.1)	28.9 (5.4)	26.9 (4.3)	27.3 (5.1)	<0.001
College or above, *n* (%)	1,339 (38.9)	151 (30.6)	251 (29.5)	277 (38.5)	288 (26.2)	<0.001
Married or partnered, *n* (%)	2,617 (75.9)	344 (69.6)	550 (64.6)	515 (71.4)	694 (63.1)	<0.001
Smoking status, *n* (%)						<0.001
Never smoker	1,452 (42.1)	167 (33.8)	284 (33.3)	252 (35.0)	257 (23.4)	
Former smoker	1,690 (49.0)	251 (50.8)	406 (47.7)	362 (50.2)	539 (49.0)	
Current smoker	308 (8.9)	76 (15.4)	162 (19.0)	107 (14.8)	303 (27.6)	
Drink less than once a week, *n* (%)	1,211 (35.1)	211 (42.7)	375 (44.1)	235 (32.7)	458 (41.7)	<0.001
Inactive physical activity, *n* (%)	466 (13.5)	100 (20.2)	271 (31.8)	103 (14.3)	283 (25.8)	<0.001
FEV_1_, (L), mean (SD)	2.73 (0.70)	2.23 (0.54)	1.72 (0.54)	2.60 (0.67)	1.42 (0.61)	<0.001
FVC, (L), mean (SD)	3.45 (0.89)	2.64 (0.65)	2.14 (0.68)	4.20 (1.15)	2.82 (0.99)	<0.001
PF, (L/min), mean (SD)	416.0 (133.7)	378.2 (126.9)	299.6 (117.2)	391.2 (139.9)	273.4 (127.5)	<0.001
CRP, (mg/L), median (IQR)	1.8 (0.8–3.6)	2.1 (1.1–4.2)	3.1 (1.5–6.1)	1.6 (0.8–3.5)	2.6 (1.2–5.4)	<0.001
Phlegm, *n* (%)	476 (13.8)	94 (19.0)	206 (24.2)	134 (18.6)	389 (35.4)	<0.001
Wheezing, *n* (%)	358 (10.4)	96 (19.4)	226 (26.5)	97 (13.5)	372 (33.8)	<0.001
Dyspnea, *n* (%)	278 (8.0)	73 (14.9)	210 (24.6)	45 (6.3)	258 (26.3)	<0.001
Chronic bronchitis/emphysema, *n* (%)	94 (2.7)	20 (4.0)	77 (9.0)	30 (4.2)	165 (15.0)	<0.001
CVD, *n* (%)	533 (15.4)	109 (22.1)	249 (29.2)	97 (13.5)	268 (24.4)	<0.001
Cancer, *n* (%)	244 (7.1)	35 (7.1)	73 (8.6)	57 (7.9)	83 (7.6)	0.629

*This description of baseline characteristics was performed among the 6,616 subjects.*

*P values were calculated using the one-way ANOVA or Kruskal–Wallis rank sum tests for continuous variables, and chi-square tests for categorical variables.*

*PRISm, Preserved Ratio Impaired Spirometry; GOLD, Global Initiative for Chronic Obstructive Lung Disease; BMI, body mass index; FEV_1_, forced expiratory volume in one second; FVC, forced vital capacity; PF, peak flow; CRP, C-reactive protein; CVD, cardio-cerebrovascular disease; SD, standard deviation; IQR, interquartile range*.

### Long-Term Mortality Risks for Different Lung Function Categories

After an average of 7.7 years of follow-up among the 6,616 subjects, 874 subjects died including 203 CVD deaths, 83 respiratory deaths, 273 cancer deaths, 120 other causes of deaths, and 195 with missing data on the cause of deaths. Table 2 shows the incidence rates and risks of all-cause mortality, respiratory mortality, and CVD mortality by the different lung function categories. Subjects with GOLD2–4 had the highest incidence rates of 37.78/1,000 person years for all-cause mortality, 6.32/1,000 for respiratory mortality, and 9.78/1,000 for CVD mortality, then followed by those with severe PRISm, and the normal spirometry group had the lowest incidence rates of these outcomes. After adjusting for the confounding factors, compared with the normal spirometry group, subjects with severe PRISm significantly increased the risks of all-cause mortality (HR = 1.91, 95%CI = 1.58–2.31), respiratory mortality (HR = 6.02, 95%CI = 2.83–12.84), and CVD mortality (HR = 2.11, 95%CI = 1.42–3.13), while these increased risks were not significant for mild PRISm. Significantly increased risks of all-cause mortality (HR = 1.85, 95%CI = 1.55–2.20), respiratory mortality (HR = 6.61, 95%CI = 3.25–13.43), and CVD mortality (HR = 2.08, 95%CI = 1.45–3.00) were also observed among subjects with GOLD2–4, whereas subjects with GOLD1 only had significantly increased risk for all-cause mortality (HR = 1.45, 95%CI = 1.15–1.83). When compared with the severe PRISm group, mild PRISm group had significantly decreased risks of all-cause mortality (HR = 0.55, 95%CI=0.41–0.74), respiratory mortality (HR = 0.26, 95%CI = 0.08–0.87), and a borderline significantly decreased risk of CVD mortality (HR = 0.59, 95%CI = 0.33–1.07). However, there was no significant difference between severe PRISm and GOLD2–4 for the risks of all-cause mortality (HR = 0.97, 95%CI = 0.80–1.17), respiratory mortality (HR = 1.10, 95%CI = 0.66–1.83), and CVD mortality (HR = 0.99, 95%CI = 0.68–1.44).

**Table 2:** Incidence rates and risks of all-cause mortality, respiratory mortality, and CVD mortality among different lung function categories.

**Outcomes**	**Event, *n***	**Incidence rate**	**HR1 (95%CI)† **	**P1 † **	**HR2 (95%CI)‡ **	**P2‡ **
**All-cause mortality**						
Normal spirometry	278	12.16	1 (reference)		0.52 (0.43–0.63)	<0.001
Mild PRISm	57	17.55	1.05 (0.79–1.40)	0.758	0.55 (0.41–0.74)	<0.001
Severe PRISm	187	36.66	1.91 (1.58–2.31)	<0.001	1 (reference)	
GOLD1	101	21.71	1.45 (1.15–1.83)	0.001	0.76 (0.59–0.97)	0.034
GOLD2-4	251	37.78	1.85 (1.55–2.20)	<0.001	0.97 (0.80–1.17)	0.771
**Respiratory mortality**						
Normal spirometry	10	0.44	1 (reference)		0.17 (0.08–0.35)	<0.001
Mild PRISm	3	0.92	1.57 (0.43–5.75)	0.504	0.26 (0.08–0.87)	0.029
Severe PRISm	24	4.70	6.02 (2.83–12.84)	<0.001	1 (reference)	
GOLD1	4	0.86	1.32 (0.41–4.25)	0.640	0.22 (0.08–0.64)	0.006
GOLD2–4	42	6.32	6.61 (3.25–13.43)	<0.001	1.10 (0.66–1.83)	0.677
**CVD mortality**						
Normal spirometry	57	2.49	1 (reference)		0.47 (0.32–0.70)	<0.001
Mild PRISm	15	4.62	1.25 (0.71–2.22)	0.449	0.59(0.33–1.07)	0.080
Severe PRISm	48	9.41	2.11 (1.42–3.13)	<0.001	1 (reference)	
GOLD1	18	3.87	1.24 (0.73–2.11)	0.425	0.59 (0.34–1.02)	0.062
GOLD2–4	65	9.78	2.08 (1.45–3.00)	<0.001	0.99 (0.68–1.44)	0.960

*This survival analysis was performed among the 6,616 subjects.*

*The incidence rate was presented as per 1,000 person-years.*

*^†^HR1 and P1 adjusted for age, sex, marital status, education level, BMI, baseline CVD and cancer, smoking status, drinking status, and physical activity level using normal spirometry as reference.*

*^‡^HR2 and P2 adjusted for the same covariates with HR1 using severe PRISm as reference.*

*PRISm, Preserved Ratio Impaired Spirometry; GOLD, Global Initiative for Chronic Obstructive Lung Disease; CVD, cardio-cerebrovascular disease; HR, hazard ratio; CI, confidence interval; P, P-value*.

### Longitudinal Transitions of Different Lung Function Categories

After an average 4-year follow-up, 4,048 subjects were included for the longitudinal transition from Wave 2 to Wave 4. Supplementary Table 1 shows the baseline characteristics of included subjects at Wave 4 (*N* = 4,048) and those who were lost to follow-up at Wave 4 (*N* = 2,568). From Wave 2 to Wave 4, different transition patterns of mild PRISm and severe PRISm are summarized in Table 3. Among the subjects with mild PRISm at Wave 2, 19.6% maintained their status and 40.2% transitioned toward normal spirometry. In addition, 22.7% of mild PRISm transitioned toward severe PRISm, whereas only a small fraction of mild PRISm subjects changed their status to GOLD1 (7.6%) or GOLD2–4 (10.0%). Mild PRISm subjects who transitioned toward normal spirometry and GOLD1 showed increases in FVC with the medians of 57.5 (IQR: 21.8 to 122.9) and 217.8 (IQR: 150.3 to 330.1) mL/year, respectively. Subjects who changed from mild PRISm to severe PRISm exhibited declines in both FEV_1_ (median: −87.0, IQR: −114.9 to −60.3, mL/year) and FVC (median: −89.3, IQR: −147.0 to −50.6, mL/year). Mild PRISm subjects who changed to GOLD2–4 exhibited the decline in FEV_1_ (median: −98.0, IQR: −140.9 to −61.2, ml/year), while the change of FVC was not consistent (median: 15.0, IQR: −45.0 to 67.5, ml/year). In contrast, 42.4% of severe PRISm subjects at Wave 2 maintained their status and 28.3% transitioned toward GOLD2–4. Only a small fraction of severe PRISm transitioned toward normal spirometry (15.2%), mild PRISm (9.0%), and GOLD1 (5.0%). Increases in FEV_1_ and FVC were observed among the severe PRISm subjects who transitioned toward normal spirometry, mild PRISm, and GOLD1. Those who changed from severe PRISm to GOLD2–4 showed a decline in FEV_1_ (median: −56.7, IQR: −88.9 to −17.9, mL/year), but an increase in FVC (median: 68.6, IQR: −0.2 to 169.6, mL/year). In addition, 2,803 subjects were included for the longitudinal transition from Wave 4 to Wave 6. The baseline characteristics of included subjects at Wave 6 (*N* = 2,803) and those who were missed to follow-up at Wave 6 (*N* = 1,245) are presented in Supplementary Table 2. Similar transition patterns of mild PRISm and severe PRISm were also observed from Wave 4 to Wave 6 (Table 4). Mild PRISm was more likely to transition toward normal spirometry (54.7%), while severe PRISm tended to maintain the status (30.4%) or transition toward GOLD2-4 (33.9%). The detailed transition patterns of other lung function categories are summarized in Supplementary Tables 3, 4.

**Table 3:** Changes of lung function categories, FEV_1_, and FVC among subjects with mild PRISm and severe PRISm from Wave 2 to Wave 4.

**Wave 2**		**Wave 4**	**N (%)**	**ΔFEV_**1**_^†^, mL/year**	**ΔFVC^†^, mL/year**
Mild PRISm	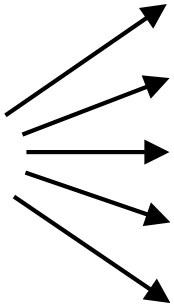	Normal spirometry	117 (40.2)	2.5 (−20.4 to 36.0)	57.5 (21.8 to 122.9)
		Mild PRISm	57 (19.6)	−29.4 (−64.0 to −7.5)	−37.5 (−62.2 to 3.2)
		Severe PRISm	66 (22.7)	−87.0 (−114.9 to −60.3)	−89.3 (−147.0 to −50.6)
		GOLD1	22 (7.6)	1.5 (−37.1 to 37.4)	217.8 (150.3 to 330.1)
		GOLD2-4	29 (10.0)	−98.0 (−140.9 to −61.2)	15.0 (−45.0 to 67.5)
Severe PRISm	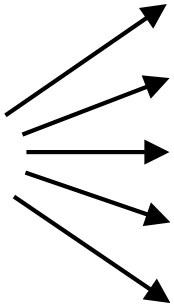	Normal spirometry	64 (15.2)	78.8 (36.9 to 179.9)	96.9 (65.8 to 233.4)
		Mild PRISm	38 (9.0)	37.6 (−8.5 to 59.4)	37.6 (−3.7 to 79.4)
		Severe PRISm	178 (42.4)	−30.6 (−58.3 to 2.9)	−24.0 (−79.6 to 14.7)
		GOLD1	21 (5.0)	104.4 (33.9 to 177.5)	339.1 (228.3 to 440.0)
		GOLD2–4	119 (28.3)	−56.7 (−88.9 to −17.9)	68.6 (−0.2 to 169.6)

*The analysis of the longitudinal transition from Wave 2 to Wave 4 was performed among the 4,048 subjects.*

*†ΔFEV_1_ and ΔFVC were calculated as the FEV_1_ and FVC at Wave 4 minus the FEV_1_ and FVC at Wave 2, respectively. Data were expressed as median (interquartile range).*

*PRISm, Preserved Ratio Impaired Spirometry; GOLD, Global Initiative for Chronic Obstructive Lung Disease; FEV_1_, forced expiratory volume in 1 s; FVC, forced vital capacity*.

**Table 4:** Changes of lung function categories, FEV_1_, and FVC among subjects with mild PRISm and severe PRISm from Wave 4 to Wave 6.

**Wave 4**		**Wave 6**	**N(%)**	**ΔFEV_**1**_†, mL/year**	**ΔFVC†, mL/year**
Mild PRISm	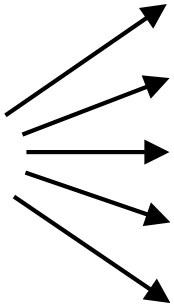	Normal spirometry	94 (54.7)	4.4 (−20.9 to 42.7)	73.8 (44.8 to 148.8)
		Mild PRISm	17 (9.9)	−43.4 (−55.4 to −34.6)	−9.3 (−21.9 to 20.2)
		Severe PRISm	16 (9.3)	−73.2 (−134.6 to −55.5)	−69.5 (−111.7 to −44.5)
		GOLD 1	17 (9.9)	−14.0 (−47.2 to 19.2)	158.8 (110.3 to 184.6)
		GOLD 2–4	28 (16.3)	−84.2 (−118.9 to −68.6)	2.6 (−54.2 to 114.5)
Severe PRISm	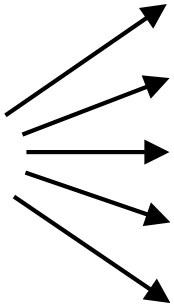	Normal spirometry	56 (19.8)	63.7 (18.1 to 233.7)	115.9 (62.1 to 315.5)
		Mild PRISm	24 (8.5)	3.3 (−22.4 to 23.8)	33.9 (−4.3 to 90.1)
		Severe PRISm	86 (30.4)	−30.3 (−57.5 to −11.2)	−10.1 (−53.5 to 37.3)
		GOLD 1	21 (7.4)	73.2 (13.9 to 201.1)	224.4 (169.3 to 480.8)
		GOLD 2–4	96 (33.9)	−38.1 (−78.3 to −1.8)	70.1 (15.9 to 163.0)

*The analysis of the longitudinal transition from Wave 4 to Wave 6 was performed among the 2,803 subjects.*

*†ΔFEV_1_ and ΔFVC were calculated as the FEV_1_ and FVC at Wave 6 minus the FEV_1_ and FVC at Wave 4, respectively. Data were expressed as median (interquartile range).*

*PRISm, Preserved Ratio Impaired Spirometry; GOLD, Global Initiative for Chronic Obstructive Lung Disease; FEV_1_, forced expiratory volume in 1 s; FVC, forced vital capacity*.

### Sensitivity Analyses

When using LLN criteria to define the lung function categories, consistent results for the baseline characteristics and long-term mortality risks were found with the fixed-threshold criteria (Supplementary Tables 5, 6). Similarly, the differences between mild PRISm and severe PRISm in the longitudinal transition patterns were consistent with the fixed-threshold criteria (Supplementary Tables 7, 8).

In the survival analyses, consistent results were found after excluding subjects with asthma at baseline (Supplementary Table 9). When stratified by sex, no significant difference was found between men and women (Supplementary Table 10). When stratified by the smoking status, severe PRISm increased long-term mortality risks among never smokers and ever smokers, while GOLD2–4 only increased these risks among ever smokers (Supplementary Table 11).

## Discussion

In the present study, two subtypes of PRISm were identified and different clinical characteristics, long-term mortality risks, and longitudinal transition patterns were found. Severe PRISm had increased respiratory symptoms, higher risks of all-cause mortality, respiratory mortality, and CVD mortality than mild PRISm. Furthermore, mild PRISm and severe PRISm showed different transition patterns during follow-up. Mild PRISm tended to transition toward normal spirometry, while severe PRISm tended to maintain the status or transition toward GOLD2–4.

Previous studies had found that PRISm was a heterogeneous phenotype (5, 6, 16). Identifying the subtypes of PRISm would allow us to perform risk stratification and individualized management of PRISm. The COPDgene study had used machine learning analyses to identify the subtypes of PRISm (28). However, this study only included moderate and heavy smokers (≥10 pack-years), and the results of machine learning were not readily interpretable, which might limit the application in clinical practices. In our study, we identified two subtypes of PRISm by the common indicators of FEV_1_ and FVC in the general population and found significant differences between the two subtypes in respiratory symptoms, the risks of all-cause mortality, respiratory mortality, and CVD mortality. Increased risks of all-cause mortality were consistently found among subjects with PRISm in previous studies (1, 2, 5). These might mainly reflect the adverse effects of severe PRISm. The Rotterdam study showed higher risks of CVD mortality in PRISm subjects with impairments of FVC (5), which was also consistent with the result of severe PRISm. Furthermore, multiple sensitivity analyses indicated that severe PRISm presented increased risks in both men and women, smokers and non-smokers, fixed-threshold and LLN criteria, but not for mild PRISm. These results confirmed the stability of risk differences between the two subtypes of PRISm.

Besides the associations of single spirometry measurement at baseline with long-term outcomes, investigating longitudinal trajectories of PRISm could elucidate the disease course and progression. Previous transition studies found that PRISm was a fluid state with a high frequency of transitioning toward different lung function categories. The Rotterdam study showed that 32.6% of PRISm transitioned toward COPD and 10.4% transitioned toward normal spirometry (5), while those were 25.1 and 22.2% in the COPDgene study, respectively (16). In our study with three repeated spirometry measurements, we found different transition patterns of mild PRISm and severe PRISm. Mild PRISm tended to transition toward normal spirometry, while severe PRISm tended to maintain the status or transition toward GOLD2–4, confirming the heterogeneity of the two subtypes. In addition, we observed the direct transitions from normal spirometry to GOLD1 or to PRISm, whereas the proportion of the transition from GOLD1 to PRISm or from PRISm to GOLD1 was relatively low. This result was consistent with the previous findings that the progression of COPD had two distinct disease courses: (1) the emphysematous-predominant pathway that subjects first developed GOLD1, then progressed to GOLD2-4; (2) the airway-disease predominant pathway that subjects first developed PRISm, then progressed to GOLD2-4 (29, 30). As a supplement for pathway 2, our transition results suggested that mild PRISm might be the early stage of PRISm and severe PRISm was the subsequent progression of mild PRISm.

Our findings have important implications in clinical and public health practices. The epidemiologic data had revealed that the proportion of PRISm was significant in the general population (1–10). However, this phenotype was highly heterogeneous, and precise stratified management of PRISm was not well addressed according to current guidelines. Our results identified two subtypes of PRISm and found severe PRISm exhibited a poor prognosis like GOLD2–4, emphasizing the importance for clinicians to focus on this abnormal lung function pattern. Comprehensive risk factor interventions, such as smoking cessation and stringent clinical treatment, should be conducted for the subjects with severe PRISm as early as possible. In the health examination, lung function tests should also be encouraged to screen for severe PRISm, not only for COPD. On the other hand, despite the similar long-term mortality risks between mild PRISm and normal spirometry, there was a portion of mild PRISm progressing to severe PRISm or GOLD2–4 during follow-up. Therefore, regular screening for lung function and early interventions would be needed to avoid further deterioration. In addition, the definitions of PRISm were inconsistent in previous studies (7, 18). Our results would be suggestive for establishing a uniform definition and severity staging for PRISm, allowing for better descriptions of this phenotype and its interpretation.

Our study had some strengths. To our knowledge, this study was the first to identify the subtypes of PRISm based on the different degrees of reductions in FEV_1_ and FVC. Furthermore, multiple pieces of evidence including the clinical characteristics, long-term mortality risks, and longitudinal transition patterns enabled a more comprehensive assessment of the differences between mild PRISm and severe PRISm. The diverse sensitivity analyses also ensured the robustness of our findings. There were several limitations in this study. First, the ELSA only performed prebronchodilator spirometry, which might affect the subjects with reversible airflow obstruction, such as asthma patients. The prebronchodilator spirometry would underestimate their actual lung functions. However, our results were consistent after excluding the subjects with asthma (Supplementary Table 9), suggesting the potential bias was small. Second, the ELSA focused on the middle-aged and elderly population without the individuals aged <50 years. This design would limit the generalizability of our findings. Further studies should be conducted on the young population in the future. Third, although we had adjusted for the smoking status, a more accurate description of the smoking exposure, such as pack-year, was not available in the ELSA. Fourth, due to the lack of relevant data, detailed pathophysiological features and mechanisms for the two subtypes of PRISm were unclear. Finally, the subjects with poor health status at baseline were more likely to drop out or die during follow-up. Therefore, there might be a healthy subject bias in the longitudinal transition analyses.

In conclusion, we identified two subtypes of PRISm and found that severe PRISm had increased respiratory symptoms, higher mortality risks, and a higher probability of progressing to GOLD2–4 than mild PRISm. These findings provided new evidence for the stratified management of individuals with PRISm.

## Data Availability Statement

Publicly available datasets were analyzed in this study. This data can be found here: www.ukdataservice.ac.uk/.

## Ethics Statement

The studies involving human participants were reviewed and approved by London Multi-Center Research Ethics Committee. The patients/participants provided their written informed consent to participate in this study.

## Author Contributions

DH, MG, and YZhu designed the analysis plan. DH and MG analyzed the data. YS, QW, ZC, JL, YZho, and KY provided the interpretation of the results. DH, MG, YS, and YZhu wrote the manuscript. DH, MG, YS, KY, and YZhu made the revision of the manuscript. All authors read and approved the final manuscript.

## Funding

This work was supported by the grant from the National Key Research and Development Program of China (2017YFC0907004) and the grant from the Major Research and Development Project of Zhejiang Province (2020C03062).

## Conflict of Interest

The authors declare that the research was conducted in the absence of any commercial or financial relationships that could be construed as a potential conflict of interest.

## Publisher's Note

All claims expressed in this article are solely those of the authors and do not necessarily represent those of their affiliated organizations, or those of the publisher, the editors and the reviewers. Any product that may be evaluated in this article, or claim that may be made by its manufacturer, is not guaranteed or endorsed by the publisher.

## Abbreviations

BMI: body mass index
CI: confidence interval
COPD: chronic obstructive pulmonary disease
CRP: C-reactive protein
CVD: cardio-cerebrovascular disease
ELSA: English Longitudinal Study of Aging
GOLD: Global Initiative for Chronic Obstructive Lung Disease
FEV_1_: forced expiratory volume in 1 s
FVC: forced vital capacity
LLN: lower limit of normal
PF: peak flow
PRISm: Preserved Ratio Impaired Spirometry
SD: standard deviation.
