# Supplementary material for: Different Risks of Mortality and Longitudinal Transition Trajectories in New Potential Subtypes of the Preserved Ratio Impaired Spirometry: Evidence From the English Longitudinal Study of Aging
Source: Front Med (Lausanne). 2021 Nov 11;8:755855. doi: 10.3389/fmed.2021.755855 (PMC8631955; doi:10.3389/fmed.2021.755855)
Supplement: Supplementary file 1 [file Data_Sheet_1.docx]

**Supplementary Table 1.** Baseline characteristics of the included subjects at Wave 4 and loss to follow-up subjects at Wave 4

|  | Included subjects at Wave 4 | Loss to follow-up  at Wave 4 | P value |
| --- | --- | --- | --- |
| Number | 4048 | 2568 |  |
| Age, year, mean(SD) | 64.6(8.3) | 65.8(9.1) | <0.001 |
| Female, n(%) | 2227(55.0) | 1382(53.8) | 0.353 |
| BMI, (kg/m2), mean(SD) | 28.0(4.8) | 28.0(4.9) | 0.577 |
| College or above, n(%) | 1572(38.8) | 745(29.0) | <0.001 |
| Married or partnered, n(%) | 2966(73.3) | 1754(68.3) | <0.001 |
| Smoking status, n(%) |  |  | <0.001 |
| Never smoker | 1548(38.2) | 864(33.6) |  |
| Former smoker | 1969(48.6) | 1289(50.2) |  |
| Current smoker | 531(13.1) | 425(16.6) |  |
| Drink less than once a week, n(%) | 1427(35.3) | 1074(41.8) | <0.001 |
| Inactive physical activity, n(%) | 560(13.8) | 663(25.8) | <0.001 |
| FEV_1_, (L), mean(SD) | 2.42(0.83) | 2.19(0.84) | <0.001 |
| FVC, (L), mean(SD) | 3.31(1.05) | 3.02(1.07) | <0.001 |
| PF, (L/min), mean(SD) | 388.2(140.9) | 345.9(142.6) | <0.001 |
| CRP, (mg/L), median(IQR) | 1.9(0.9-3.9) | 2.0(1.0-4.3) | <0.001 |
| Phlegm, n(%) | 727(18.0) | 573(22.3) | <0.001 |
| Wheezing, n(%) | 629(15.5) | 521(20.3) | <0.001 |
| Dyspnea, n(%) | 1179(29.1) | 972(37.9) | <0.001 |
| Chronic bronchitis/emphysema, n(%) | 179(4.4) | 207(8.1) | <0.001 |
| CVD, n(%) | 622(15.4) | 634(24.7) | <0.001 |
| Cancer, n(%) | 265(6.6) | 227(8.8) | 0.895 |

PRISm: Preserved Ratio Impaired Spirometry; GOLD: Global Initiative for Chronic Obstructive Lung Disease; BMI: body mass index; FEV_1_: forced expiratory volume in one second; FVC: forced vital capacity; PF: peak flow; CRP: C-reactive protein; CVD: cardio-cerebrovascular disease; SD: standard deviation; IQR: interquartile range

**Supplementary Table 2.** Baseline characteristics of the included subjects at Wave 6 and loss to follow-up subjects at Wave 6

|  | Included subjects at Wave 6 | Loss to follow-up  at Wave 6 | P value |
| --- | --- | --- | --- |
| Number | 2803 | 1245 |  |
| Age, year, mean(SD) | 63.4(7.6) | 67.3(9.2) | <0.001 |
| Female, n(%) | 1533(54.7) | 694(55.7) | 0.558 |
| BMI, (kg/m2), mean(SD) | 27.9(4.7) | 28.1(5.1) | 0.335 |
| College or above, n(%) | 1163(41.5) | 409(32.9) | <0.001 |
| Married or partnered, n(%) | 2112(75.4) | 854(68.6) | <0.001 |
| Smoking status, n(%) |  |  | 0.024 |
| Never smoker | 1102(39.3) | 446(35.8) |  |
| Former smoker | 1356(48.4) | 613(49.2) |  |
| Current smoker | 345(12.3) | 186(14.9) |  |
| Drink less than once a week, n(%) | 936(33.4) | 491(39.5) | <0.001 |
| Inactive physical activity, n(%) | 337(12.0) | 223(17.9) | <0.001 |
| FEV_1_, (L), mean(SD) | 2.50(0.83) | 2.25(0.80) | <0.001 |
| FVC, (L), mean(SD) | 3.40(1.04) | 3.10(1.02) | <0.001 |
| PF, (L/min), mean(SD) | 401.9(140.5) | 357.4(136.9) | <0.001 |
| CRP, (mg/L), median(IQR) | 1.8(0.9-3.7) | 1.9(1.0-4.4) | 0.009 |
| Phlegm, n(%) | 460(16.4) | 267(21.5) | <0.001 |
| Wheezing, n(%) | 405(14.5) | 224(18.0) | 0.005 |
| Dyspnea, n(%) | 755(26.9) | 424(34.1) | <0.001 |
| Chronic bronchitis/emphysema, n(%) | 102(3.6) | 77(6.2) | <0.001 |
| CVD, n(%) | 389(13.9) | 233(18.7) | <0.001 |
| Cancer, n(%) | 165(5.9) | 100(8.0) | 0.013 |

PRISm: Preserved Ratio Impaired Spirometry; GOLD: Global Initiative for Chronic Obstructive Lung Disease; BMI: body mass index; FEV_1_: forced expiratory volume in one second; FVC: forced vital capacity; PF: peak flow; CRP: C-reactive protein; CVD: cardio-cerebrovascular disease; SD: standard deviation; IQR: interquartile range

**Supplementary Table 3.** Changes of lung function categories, FEV_1_, and FVC among subjects with normal spirometry, GOLD1 and GOLD2-4 from Wave 2 to Wave 4

| Wave 2 | Wave 4 | N(%) | △FEV_1_†, ml/year | △FVC†, ml/year |
| --- | --- | --- | --- | --- |
|  | Normal spirometry | 1664(73.0) | -33.9(-67.4 to -2.4) | -40.0(-93.9 to 15.7) |
| Normal  spirometry | Mild PRISm | 116(5.1) | -80.4(-110.4 to -40.0) | -125.2(-181.4 to -84.9) |
|  | Severe PRISm | 116(5.1) | -131.9(-212.7 to -90.5) | -190.5(-301.7 to -132.9) |
|  | GOLD1 | 281(12.3) | -36.7(-65.7 to -7.1) | 102.1(5.6 to 271.8) |
|  | GOLD2-4 | 103(4.5) | -245.0(-412.1 to -142.9) | -58.9(-111.6 to 12.4) |
|  | Normal spirometry | 221(47.8) | -26.3(-62.6 to 11.8) | -226.7(-368.0 to -112.2) |
|  | Mild PRISm | 14(3.0) | -52.9(-100.8 to -45.9) | -323.0(-550.5 to -260.5) |
| GOLD1 | Severe PRISm | 19(4.1) | -80.9(-134.5 to -67.5) | -330.6(-544.3 to -232.1) |
|  | GOLD1 | 156(33.8) | -38.3(-66.0 to -4.1) | -43.0(-104.7 to 42.2) |
|  | GOLD2-4 | 52(11.3) | -128.2(-268.0 to -97.1) | -197.4(-337.8 to -110.8) |
|  | Normal spirometry | 115(19.3) | 317.5(158.6 to 454.3) | -22.5(-71.0 to 34.6) |
|  | Mild PRISm | 30(5.0) | 150.2(44.8 to 297.5) | -140.6(-276.7 to -47.5) |
| GOLD2-4 | Severe PRISm | 84(14.1) | 0.0(-49.9 to 64.8) | -96.5(-241.3 to -18.4) |
|  | GOLD1 | 49(8.2) | 219.6(60.0 to 378.8) | 62.4(-19.6 to 128.6) |
|  | GOLD2-4 | 317(53.3) | -32.0(-72.0 to 10.7) | -37.3(-122.5 to 42.4) |

†△FEV_1_ and △FVC were calculated as the FEV_1_ and FVC at Wave 4 minus the FEV_1_ and FVC at Wave 2, respectively. Data were expressed as median (interquartile range).

PRISm: Preserved Ratio Impaired Spirometry; GOLD: Global Initiative for Chronic Obstructive Lung Disease; FEV_1_: forced expiratory volume in one second; FVC: forced vital capacity

**Supplementary Table 4.** Changes of lung function categories, FEV_1_, and FVC among subjects with normal spirometry, GOLD1 and GOLD2-4 from Wave 4 to Wave 6

| Wave 4 | Wave 6 | N(%) | △FEV_1_†, ml/year | △FVC†, ml/year |
| --- | --- | --- | --- | --- |
|  | Normal spirometry | 1161(73.4) | -33.1(-68.8 to -1.1) | -13.1(-63.1 to 42.1) |
| Normal  spirometry | Mild PRISm | 38(2.4) | -81.9(-108.9 to -56.5) | -99.1(-161.2 to -62.9) |
|  | Severe PRISm | 43(2.7) | -119.2(-163.2 to -86.9) | -146.3(-224.4 to -95.3) |
|  | GOLD1 | 269(17.0) | -47.5(-76.5 to -13.1) | 55.2(6.7 to 108.4) |
|  | GOLD2-4 | 70(4.4) | -124.8(-194.7 to -84.6) | -53.6(-128.5 to 5.5) |
|  | Normal spirometry | 156(40.5) | -33.7(-71.7 to 7.9) | -222.9(-381.5 to -109.3) |
|  | Mild PRISm | 5(1.3) | -56.2(-82.8 to -38.3) | -223.3(-254.4 to -80.1) |
| GOLD1 | Severe PRISm | 6(1.6) | -288.7(-330.1 to -165.5) | -645.1(-805.5 to -419.9) |
|  | GOLD1 | 172(44.7) | -43.8(-75.3 to -13.2) | -57.7(-141.6 to 10.7) |
|  | GOLD2-4 | 46(11.9) | -101.1(-151.1 to -82.6) | -144.3(-237.4 to -71.7) |
|  | Normal spirometry | 49(12.8) | 266.4(157.1 to 405.1) | -31.2(-114.5 to 73.6) |
|  | Mild PRISm | 6(1.6) | 204.9(31.8 to 523.9) | -93.6(-160.4 to -57.0) |
| GOLD2-4 | Severe PRISm | 21(5.5) | -21.9(-49.3 to 8.0) | -137.6(-241.2 to -86.2) |
|  | GOLD1 | 45(11.8) | 59.7(21.7 to 131.6) | 50.6(-14.2 to 98.8) |
|  | GOLD2-4 | 261(68.3) | -33.5(-68.0 to 8.9) | -21.0(-88.8 to 65.0) |

†△FEV_1_ and △FVC were calculated as the FEV_1_ and FVC at Wave 6 minus the FEV_1_ and FVC at Wave 4, respectively. Data were expressed as median (interquartile range).

PRISm: Preserved Ratio Impaired Spirometry; GOLD: Global Initiative for Chronic Obstructive Lung Disease; FEV_1_: forced expiratory volume in one second; FVC: forced vital capacity

**Supplementary Table 5.** Baseline characteristics among subjects by the lung function categories based on the LLN criteria

|  | Normal spirometry | Mild PRISm | Severe PRISm | GOLD1 | GOLD2-4 | P value |
| --- | --- | --- | --- | --- | --- | --- |
| Number | 4366 | 421 | 646 | 469 | 714 |  |
| Age, year, mean(SD) | 65.3(8.9) | 66.3(8.9) | 67.6(9.5) | 65.2(8.9) | 67.4(9.3) | <0.001 |
| Female, n(%) | 2411(55.2) | 211(50.1) | 358(55.4) | 254(54.2) | 375(52.5) | 0.024 |
| BMI, (kg/m2), mean(SD) | 28.0(4.7) | 28.9(5.1) | 29.0(5.7) | 27.0(4.5) | 27.2(5.2) | <0.001 |
| College or above, n(%) | 1654(37.9) | 122(29.0) | 181(28.1) | 164(34.9) | 185(26.0) | <0.001 |
| Married or partnered, n(%) | 3242(74.3) | 285(67.7) | 414(64.1) | 320(68.2) | 459(64.3) | <0.001 |
| Smoking status, n(%) |  |  |  |  |  | <0.001 |
| Never smoker | 1766(40.4) | 138(32.8) | 187(28.9) | 147(31.3) | 174(24.4) |  |
| Former smoker | 2160(49.5) | 203(48.2) | 322(49.8) | 228(48.6) | 335(46.9) |  |
| Current smoker | 440(10.1) | 80(19.0) | 137(21.2) | 94(20.0) | 205(28.7) |  |
| Drink less than once a week, n(%) | 1567(35.9) | 162(38.5) | 293(45.3) | 162(34.5) | 306(42.9) | <0.001 |
| Inactive physical activity, n(%) | 646(14.8) | 99(23.5) | 227(35.1) | 70(14.9) | 181(25.4) | <0.001 |
| FEV_1_, (L), mean(SD) | 2.64(0.72) | 2.10(0.53) | 1.56(0.52) | 2.47(0.66) | 1.22(0.55) | <0.001 |
| FVC, (L), mean(SD) | 3.38(0.93) | 2.54(0.62) | 2.00(0.67) | 4.29(1.18) | 2.83(1.02) | <0.001 |
| PF, (L/min), mean(SD) | 405.2(135.7) | 354.9(125.7) | 275.3(115.7) | 367.2(138.0) | 268.0(130.3) | <0.001 |
| CRP, (mg/L), median(IQR) | 1.8(0.9-3.7) | 2.7(1.3-5.6) | 3.3(1.7-6.5) | 1.7(0.8-3.9) | 2.5(1.2-4.9) | <0.001 |
| Phlegm, n(%) | 670(15.3) | 94(22.3) | 186(28.8) | 100(21.3) | 249(34.9) | <0.001 |
| Wheezing, n(%) | 532(12.2) | 89(21.1) | 203(31.4) | 69(14.7) | 256(35.9) | <0.001 |
| Dyspnea, n(%) | 387(8.8) | 87(20.8) | 179(27.6) | 29(6.2) | 182(25.5) | <0.001 |
| Chronic bronchitis/emphysema, n(%) | 139(3.2) | 32(7.6) | 75(11.6) | 23(4.9) | 117(16.4) | <0.001 |
| CVD, n(%) | 731(16.7) | 117(27.8) | 175(27.1) | 65(13.9) | 168(23.5) | <0.001 |
| Cancer, n(%) | 316(7.2) | 28(6.7) | 59(9.1) | 36(7.7) | 53(7.4) | 0.498 |

P values were calculated using the one-way ANOVA or Kruskal–Wallis rank sum tests for continuous variables, and chi-square tests for categorical variables.

LLN: lower limit of normal; PRISm: Preserved Ratio Impaired Spirometry; GOLD: Global Initiative for Chronic Obstructive Lung Disease; BMI: body mass index; FEV_1_: forced expiratory volume in one second; FVC: forced vital capacity; PF: peak flow; CVD: cardio-cerebrovascular disease; CRP: C-reactive protein;

**Supplementary Table 6.** Incidence rates and risks of all-cause mortality, respiratory mortality and CVD mortality by the lung function categories based on the LLN criteria

| Outcomes | Event, n | Incidence rate | HR1(95%CI) † | P1 † | HR2(95%CI) ‡ | P2 ‡ |
| --- | --- | --- | --- | --- | --- | --- |
| All-cause mortality |  |  |  |  |  |  |
| Normal spirometry | 436 | 15.22 | 1(reference) |  | 0.53(0.44-0.64) | <0.001 |
| Mild PRISm | 64 | 24.45 | 1.35(1.04-1.76) | 0.027 | 0.72(0.54-0.96) | 0.028 |
| Severe PRISm | 154 | 40.00 | 1.88(1.56-2.27) | <0.001 | 1(reference) |  |
| GOLD1 | 62 | 20.25 | 1.34(1.02-1.75) | 0.033 | 0.71(0.53-0.96) | 0.028 |
| GOLD2-4 | 158 | 36.40 | 1.69(1.40-2.04) | <0.001 | 0.90(0.72-1.13) | 0.381 |
| Respiratory mortality |  |  |  |  |  |  |
| Normal spirometry | 19 | 0.66 | 1(reference) |  | 0.16(0.09-0.30) | <0.001 |
| Mild PRISm | 10 | 3.82 | 4.51 (2.09-9.76) | <0.001 | 0.73(0.34-1.55) | 0.422 |
| Severe PRISm | 23 | 5.97 | 6.18(3.31-11.53) | <0.001 | 1(reference) |  |
| GOLD1 | 1 | 0.33 | 0.45(0.06-3.40) | 0.441 | 0.07(0.01-0.55) | 0.011 |
| GOLD2-4 | 30 | 6.91 | 5.86(3.23-10.62) | <0.001 | 0.95(0.54-1.66) | 0.905 |
| CVD mortality |  |  |  |  |  |  |
| Normal spirometry | 99 | 3.46 | 1(reference) |  | 0.50(0.34-0.73) | <0.001 |
| Mild PRISm | 11 | 4.20 | 0.87(0.46-1.63) | 0.668 | 0.44(0.22-0.85) | 0.016 |
| Severe PRISm | 39 | 10.13 | 2.00(1.37-2.92) | <0.001 | 1(reference) |  |
| GOLD1 | 15 | 4.90 | 1.52(0.88-2.64) | 0.132 | 0.76(0.42-1.40) | 0.389 |
| GOLD2-4 | 39 | 8.98 | 1.79(1.22-2.61) | 0.003 | 0.89(0.57-1.41) | 0.630 |

The incidence rate was presented as per 1,000 person-years.

† HR1 and P1 adjusted for age, sex, marital status, education level, BMI, baseline CVD and cancer, smoking status, drinking status and physical activity level using normal spirometry as reference.

‡ HR2 and P2 adjusted for the same covariates with HR1 using severe PRISm as reference.

LLN: lower limit of normal; PRISm: Preserved Ratio Impaired Spirometry; GOLD: Global Initiative for Chronic Obstructive Lung Disease; CVD: cardio-cerebrovascular disease; HR: hazard ratio; CI: confidence interval; P: P value

**Supplementary Table 7.** Changes of lung function categories, FEV_1_, and FVC among subjects with different lung function categories from Wave 2 to Wave 4 based on the LLN criteria

| Wave 2 | Wave 4 | N(%) | △FEV_1_†, ml/year | △FVC†, ml/year |
| --- | --- | --- | --- | --- |
|  | Normal spirometry | 2303(82.2) | -34.0(-69.2 to -2.6) | -40.0(-96.5 to 19.2) |
|  | Mild PRISm | 109(3.9) | -88.8(-134.6 to -61.2) | -146.1(-225.0 to -82.5) |
| Normal | Severe PRISm | 96(3.4) | -154.0(-260.6 to -102.4) | -215.5(-344.8 to -133.4) |
| spirometry | GOLD1 | 188(6.7) | -29.4(-62.5 to 2.5) | 214.5(85.2 to 349.7) |
|  | GOLD2-4 | 104(3.7) | -273.3(-426.6 to -147.6) | -60.0(-124.9 to 24.0) |
|  | Normal spirometry | 112(47.1) | 4.7(-23.1 to 46.3) | 68.8(30.2 to 126.4) |
|  | Mild PRISm | 43(18.1) | -32.9(-53.6 to -18.5) | -38.1(-63.3 to -2.6) |
| Mild PRISm | Severe PRISm | 43(18.1) | -88.0(-112.6 to -59.4) | -106.2(-186.6 to -41.9) |
|  | GOLD1 | 14(5.9) | -15.4(-47.7 to 31.3) | 266.5(172.7 to 406.5) |
|  | GOLD2-4 | 26(10.9) | -93.3(-227.2 to -55.7) | 28.2(-38.0 to 126.0) |
|  | Normal spirometry | 61(19.4) | 122.7(43.4 to 223.6) | 167.5(88.3 to 338.4) |
|  | Mild PRISm | 36(11.5) | 19.0(-7.5 to 61.6) | 20.8(-3.1 to 61.3) |
| Severe PRISm | Severe PRISm | 132(42.0) | -26.1(-58.1 to 2.2) | -21.0(-72.1 to 22.0) |
|  | GOLD1 | 14(4.5) | 118.7(35.7 to 215.5) | 375.4(318.1 to 573.7) |
|  | GOLD2-4 | 71(22.6) | -40.8(-84.8 to -8.8) | 94.3(41.6 to 206.8) |
|  | Normal spirometry | 197(66.8) | -35.3(-66.0 to 17.9) | -300.0(-424.0 to -157.9) |
|  | Mild PRISm | 11(3.7) | -81.7(-101.2 to -60.8) | -336.6(-474.7 to -234.9) |
| GOLD1 | Severe PRISm | 10(3.4) | -136.1(-246.8 to -89.6) | -374.9(-510.7 to -276.1) |
|  | GOLD1 | 58(19.7) | -38.8(-73.1 to -8.9) | -40.0(-127.8 to 23.9) |
|  | GOLD2-4 | 19(6.4) | -206.0(-392.7 to -120.0) | -143.0(-249.9 to -91.5) |
|  | Normal spirometry | 148(36.9) | 316.3(176.5 to 461.1) | -19.6(-70.7 to 34.3) |
|  | Mild PRISm | 23(5.7) | 35.0(1.5 to 205.8) | -106.7(-232.2 to -39.6) |
| GOLD2-4 | Severe PRISm | 48(12.0) | -8.9(-81.5 to 57.6) | -142.2(-263.8 to -43.5) |
|  | GOLD1 | 24(6.0) | 140.3(48.1 to 368.5) | 166.8(20.6 to 267.1) |
|  | GOLD2-4 | 158(39.4) | -34.8(-71.9 to 11.2) | -43.6(-126.4 to 36.5) |

† △FEV_1_ and △FVC were calculated as the FEV_1_ and FVC at Wave 4 minus the FEV_1_ and FVC at Wave 2, respectively. Data were expressed as median (interquartile range).

LLN: lower limit of normal; PRISm: Preserved Ratio Impaired Spirometry; GOLD: Global Initiative for Chronic Obstructive Lung Disease; FEV_1_: forced expiratory volume in one second; FVC: forced vital capacity

**Supplementary Table 8.** Changes of lung function categories, FEV_1_, and FVC among subjects with different lung function categories from Wave 4 to Wave 6 based on the LLN criteria

| Wave 4 | Wave 6 | N(%) | △FEV_1_†, ml/year | △FVC†, ml/year |
| --- | --- | --- | --- | --- |
|  | Normal spirometry | 1785(88.9) | -36.0(-72.7 to -2.0) | -8.6(-65.6 to 51.7) |
|  | Mild PRISm | 43(2.1) | -98.4(-131.8 to -68.7) | -97.6(-172.4 to -58.1) |
| Normal | Severe PRISm | 25(1.2) | -194.6(-334.7 to -131.1) | -239.9(-389.5 to -156.4) |
| spirometry | GOLD1 | 118(5.9) | -59.0(-89.4 to -20.0) | 54.6(8.0 to 154.6) |
|  | GOLD2-4 | 36(1.8) | -142.5(-259.1 to -82.6) | -56.3(-129.3 to 4.0) |
|  | Normal spirometry | 85(59.4) | 6.4(-25.6 to 48.0) | 98.1(51.9 to 172.6) |
|  | Mild PRISm | 14(9.8) | -46.0(-59.5 to -37.8) | -34.8(-53.7 to 35.8) |
| Mild PRISm | Severe PRISm | 12(8.4) | -99.4(-124.0 to -56.9) | -77.4(-105.2 to -43.8) |
|  | GOLD1 | 5(3.5) | 17.1(2.8 to 105.1) | 234.0(162.7 to 309.1) |
|  | GOLD2-4 | 27(18.9) | -92.0(-117.8 to -37.8) | 24.4(-43.9 to 70.2) |
|  | Normal spirometry | 57(28.8) | 92.4(31.6 to 278.8) | 222.9(105.1 to 393.1) |
|  | Mild PRISm | 16(8.1) | -19.2(-26.2 to 6.1) | 51.0(16.7 to 68.5) |
| Severe PRISm | Severe PRISm | 62(31.3) | -35.1(-79.0 to -10.2) | -14.7(-71.7 to 32.8) |
|  | GOLD1 | 9(4.5) | 44.2(38.5 to 149.4) | 433.2(175.4 to 499.5) |
|  | GOLD2-4 | 54(27.3) | -42.4(-79.5 to -10.9) | 102.7(42.1 to 172.4) |
|  | Normal spirometry | 139(62.9) | -40.1(-73.0 to 4.8) | -285.5(-447.6 to -165.8) |
|  | Mild PRISm | 5(2.3) | -117.3(-132.3 to -83.7) | -257.2(-366.2 to -242.3) |
| GOLD1 | Severe PRISm | 3(1.4) | -158.2(-544.0 to -135.5) | -500.0(-1125.0 to -491.4) |
|  | GOLD1 | 60(27.1) | -40.1(-71.3 to -18.2) | -81.9(-195.0 to -16.4) |
|  | GOLD2-4 | 14(6.3) | -91.0(-125.0 to -72.5) | -120.2(-235.6 to -53.4) |
|  | Normal spirometry | 66(28.2) | 257.5(131.8 to 397.2) | 5.4(-82.0 to 66.2) |
|  | Mild PRISm | 14(6.0) | 3.5(-27.5 to 33.3) | -164.5(-430.7 to -95.2) |
| GOLD2-4 | Severe PRISm | 9(3.8) | 47.4(-12.3 to 70.9) | -170.6(-262.8 to -21.5) |
|  | GOLD1 | 16(6.8) | 70.9(12.4 to 139.1) | 62.8(-1.2 to 153.4) |
|  | GOLD2-4 | 129(55.1) | -31.5(-66.1 to 9.9) | -13.6(-87.5 to 65.9) |

† △FEV_1_ and △FVC were calculated as the FEV_1_ and FVC at Wave 6 minus the FEV_1_ and FVC at Wave 4, respectively. Data were expressed as median (interquartile range).

LLN: lower limit of normal; PRISm: Preserved Ratio Impaired Spirometry; GOLD: Global Initiative for Chronic Obstructive Lung Disease; FEV_1_: forced expiratory volume in one second; FVC: forced vital capacity

**Supplementary Table 9.** Incidence rates and risks of all-cause mortality, respiratory mortality and CVD mortality by the lung function categories after excluding the subjects with asthma

| Outcomes | Event, n | Incidence rate | HR1(95%CI) † | P1 † | HR2(95%CI) ‡ | P2 ‡ |
| --- | --- | --- | --- | --- | --- | --- |
| All-cause mortality |  |  |  |  |  |  |
| Normal spirometry | 262 | 12.35 | 1(reference) |  | 0.50(0.41-0.61) | <0.001 |
| Mild PRISm | 50 | 17.97 | 1.04(0.77-1.42) | 0.805 | 0.52(0.38-0.72) | <0.001 |
| Severe PRISm | 167 | 39.08 | 1.99(1.63-2.44) | <0.001 | 1(reference) |  |
| GOLD1 | 97 | 23.94 | 1.53(1.21-1.93) | <0.001 | 0.77(0.59-0.99) | 0.047 |
| GOLD2-4 | 200 | 39.40 | 1.88(1.55-2.27) | <0.001 | 0.94(0.76-1.16) | 0.596 |
| Respiratory mortality |  |  |  |  |  |  |
| Normal spirometry | 9 | 0.42 | 1(reference) |  | 0.15(0.07-0.34) | <0.001 |
| Mild PRISm | 3 | 1.08 | 1.92(0.52-7.15) | 0.343 | 0.29(0.09-1.00) | 0.050 |
| Severe PRISm | 21 | 4.91 | 6.51(2.91-14.56) | <0.001 | 1(reference) |  |
| GOLD1 | 4 | 0.99 | 1.55(0.47-5.07) | 0.477 | 0.24(0.08-0.70) | 0.010 |
| GOLD2-4 | 31 | 6.11 | 6.68(3.11-14.35) | <0.001 | 1.03(0.58-1.81) | 0.867 |
| CVD mortality |  |  |  |  |  |  |
| Normal spirometry | 57 | 2.69 | 1(reference) |  | 0.48(0.32,0.73) | <0.001 |
| Mild PRISm | 14 | 5.03 | 1.20(0.67-2.17) | 0.552 | 0.58(0.31,1.06) | 0.078 |
| Severe PRISm | 43 | 10.06 | 2.08(1.38-3.13) | <0.001 | 1(reference) |  |
| GOLD1 | 16 | 3.95 | 1.14(0.65-1.99) | 0.642 | 0.55(0.31,0.99) | 0.047 |
| GOLD2-4 | 55 | 10.83 | 2.08(1.42-3.05) | <0.001 | 1.01(0.67,1.51) | 0.975 |

The incidence rate was presented as per 1,000 person-years.

† HR1 and P1 adjusted for age, sex, marital status, education level, BMI, baseline CVD and cancer, smoking status, drinking status and physical activity level using normal spirometry as reference.

‡ HR2 and P2 adjusted for the same covariates with HR1 using severe PRISm as reference.

PRISm: Preserved Ratio Impaired Spirometry; GOLD: Global Initiative for Chronic Obstructive Lung Disease; CVD: cardio-cerebrovascular disease; HR: hazard ratio; CI: confidence interval; P: P value

**Supplementary Table 10.** Risks of all-cause mortality, respiratory mortality and CVD mortality among males and females by the lung function categories

| Outcomes | Males | | | | Females | | | |
| --- | --- | --- | --- | --- | --- | --- | --- | --- |
|  | HR1 (95% CI) † | P1 † | HR2 (95% CI) ‡ | P2 ‡ | HR1 (95% CI) † | P1 † | HR2 (95% CI) ‡ | P2 ‡ |
| All-cause mortality |  |  |  |  |  |  |  |  |
| Normal spirometry | 1(reference) |  | 0.50(0.38,0.66) | <0.001 | 1(reference) |  | 0.53(0.40-0.70) | <0.001 |
| Mild PRISm | 1.05(0.70,1.59) | 0.826 | 0.53(0.34,0.81) | 0.004 | 1.01(0.68-1.52) | 0.959 | 0.54(0.35,0.82) | 0.003 |
| Severe PRISm | 1.99(1.52,2.60) | <0.001 | 1(reference) |  | 1.88(1.43-2.48) | <0.001 | 1(reference) |  |
| GOLD1 | 1.79(1.33,2.39) | <0.001 | 0.90(0.65,1.24) | 0.517 | 1.04(0.71-1.53) | 0.802 | 0.55(0.37,0.82) | 0.004 |
| GOLD2-4 | 1.93(1.52,2.45) | <0.001 | 0.97(0.74,1.26) | 0.846 | 1.73(1.33-2.26) | <0.001 | 0.92(0.69,1.22) | 0.574 |
| Respiratory mortality |  |  |  |  |  |  |  |  |
| Normal spirometry | 1(reference) |  | 0.45(0.26,0.79) | <0.001 | 1(reference) |  | 0.16(0.04,0.66) | 0.010 |
| Mild PRISm | 0.93(0.11-7.65) | 0.929 | 0.37(0.13,1.07) | 0.069 | 2.91(0.47-18.01) | 0.254 | 0.48(0.10,2.36) | 0.354 |
| Severe PRISm | 6.13(2.47-15.21) | <0.001 | 1(reference) |  | 6.08(1.52-24.29) | 0.010 | 1(reference) |  |
| GOLD1 | 1.97(0.57-6.82) | 0.297 | 1.05(0.54,2.03) | 0.047 | N/A | N/A | N/A | N/A |
| GOLD2-4 | 6.03(2.57-14.16) | <0.001 | 1.05(0.62,1.77) | 0.958 | 8.87(2.43-32.44) | <0.001 | 1.46(0.57,3.71) | 0.432 |
| CVD mortality |  |  |  |  |  |  |  |  |
| Normal spirometry | 1(reference) |  | 0.16(0.07,0.41) | 0.006 | 1(reference) |  | 0.51(0.28,0.92) | 0.023 |
| Mild PRISm | 0.81(0.28-2.30) | 0.687 | 0.15(0.02,1.16) | 0.070 | 1.47(0.71-3.05) | 0.293 | 0.75(0.36,1.57) | 0.447 |
| Severe PRISm | 2.21(1.27-3.84) | 0.006 | 1(reference) |  | 1.96(1.09-3.51) | 0.023 | 1(reference) |  |
| GOLD1 | 2.32(1.26-4.27) | 0.007 | 0.32(0.10,0.98) | 0.861 | 0.24(0.06-1.02) | 0.054 | 0.12(0.03,0.52) | 0.005 |
| GOLD2-4 | 2.32(1.42-3.78) | <0.001 | 0.98(0.52,1.85) | 0.826 | 1.75(0.99-3.07) | 0.051 | 0.89(0.51,1.57) | 0.692 |

N/A indicated that there was no incident outcome in this lung function category

† HR1 and P1 adjusted for age, marital status, education level, BMI, baseline CVD and cancer, smoking status, drinking status and physical activity level using normal spirometry as reference.

‡ HR2 and P2 adjusted for the same covariates with HR1 using severe PRISm as reference.

PRISm: Preserved Ratio Impaired Spirometry; GOLD: Global Initiative for Chronic Obstructive Lung Disease; CVD: cardio-cerebrovascular disease; HR: hazard ratio; CI: confidence interval; P: P value

**Supplementary Table 11.** Risks of all-cause mortality, respiratory mortality, and CVD mortality among never smokers and ever smokers by the lung function categories

| Outcomes | Never smokers | | | | Ever smokers | | | |
| --- | --- | --- | --- | --- | --- | --- | --- | --- |
|  | HR1 (95% CI) † | P1 † | HR2 (95% CI) ‡ | P2 ‡ | HR1 (95% CI) † | P1 † | HR2 (95% CI) ‡ | P2 ‡ |
| All-cause mortality |  |  |  |  |  |  |  |  |
| Normal spirometry | 1(reference) |  | 0.55(0.38,0.79) | 0.001 | 1(reference) |  | 0.49(0.39,0.62) | <0.001 |
| Mild PRISm | 0.85(0.47-1.53) | 0.592 | 0.46(0.25,0.87) | 0.017 | 1.14(0.82-1.59) | 0.518 | 0.56(0.4,0.79) | 0.001 |
| Severe PRISm | 1.82(1.27-2.60) | 0.001 | 1(reference) |  | 2.03(1.62-2.54) | <0.001 | 1(reference) |  |
| GOLD1 | 1.44(0.95-2.18) | 0.073 | 0.79(0.50,1.26) | 0.354 | 1.45(1.10-1.92) | 0.013 | 0.72(0.53,0.96) | 0.036 |
| GOLD2-4 | 1.12(0.76-1.65) | 0.565 | 0.61(0.40,0.94) | 0.027 | 2.19(1.79-2.68) | <0.001 | 1.08(0.87,1.34) | 0.522 |
| Respiratory mortality |  |  |  |  |  |  |  |  |
| Normal spirometry | 1(reference) |  | 0.09(0.02,0.43) | 0.003 | 1(reference) |  | 0.21(0.09,0.50) | <0.001 |
| Mild PRISm | 5.15(0.70-38.08) | 0.104 | 0.44(0.08,2.27) | 0.314 | 0.62(0.08-5.01) | 0.638 | 0.13(0.02,0.99) | 0.051 |
| Severe PRISm | 11.76(2.31-59.90) | 0.003 | 1(reference) |  | 4.76(2.02-11.26) | <0.001 | 1(reference) |  |
| GOLD1 | N/A | N/A | N/A | N/A | 1.56(0.47-5.24) | 0.497 | 0.33(0.11,0.99) | 0.053 |
| GOLD2-4 | 3.52(0.60-20.73) | 0.167 | 0.30(0.08,1.16) | 0.073 | 6.86(3.14-14.97) | <0.001 | 1.44(0.80,2.59) | 0.200 |
| CVD mortality |  |  |  |  |  |  |  |  |
| Normal spirometry | 1(reference) |  | 0.64(0.29,1.37) | 0.241 | 1(reference) |  | 0.40(0.25,0.64) | <0.001 |
| Mild PRISm | 0.51(0.12-2.26) | 0.384 | 0.32(0.07,1.52) | 0.155 | 1.66(0.87-3.15) | 0.128 | 0.66(0.35,1.26) | 0.225 |
| Severe PRISm | 1.57(0.73-3.39) | 0.241 | 1(reference) |  | 2.50(1.57-3.99) | <0.001 | 1(reference) |  |
| GOLD1 | 0.78(0.26-2.36) | 0.682 | 0.50(0.15,1.61) | 0.252 | 1.46(0.78-2.71) | 0.231 | 0.58(0.31,1.09) | 0.110 |
| GOLD2-4 | 1.17(0.54-2.52) | 0.680 | 0.74(0.31,1.77) | 0.505 | 2.58(1.68-3.95) | <0.001 | 1.03(0.67,1.58) | 0.871 |

N/A indicated that there was no incident outcome in this lung function category

Ever smokers included former smokers and current smokers

† HR1 and P1 adjusted for age, sex, marital status, education level, BMI, baseline CVD and cancer, drinking status and physical activity level using normal spirometry as reference.

‡ HR2 and P2 adjusted for the same covariates with HR1 using severe PRISm as reference.

PRISm: Preserved Ratio Impaired Spirometry; GOLD: Global Initiative for Chronic Obstructive Lung Disease; CVD: cardio-cerebrovascular disease; HR: hazard ratio; CI: confidence interval; P: P value
